# Supplementary material for: The causal role of circulating inflammatory markers in osteoporosis: a bidirectional Mendelian randomized study
Source: Front Immunol. 2024 Jul 18;15:1412298. doi: 10.3389/fimmu.2024.1412298 (PMC11291241; doi:10.3389/fimmu.2024.1412298)
Supplement: Supplementary file 1 [file DataSheet_1.zip › supplementary material/CIM-OSTEOPOROSIS /GCST90274806.csv_forest.pdf]

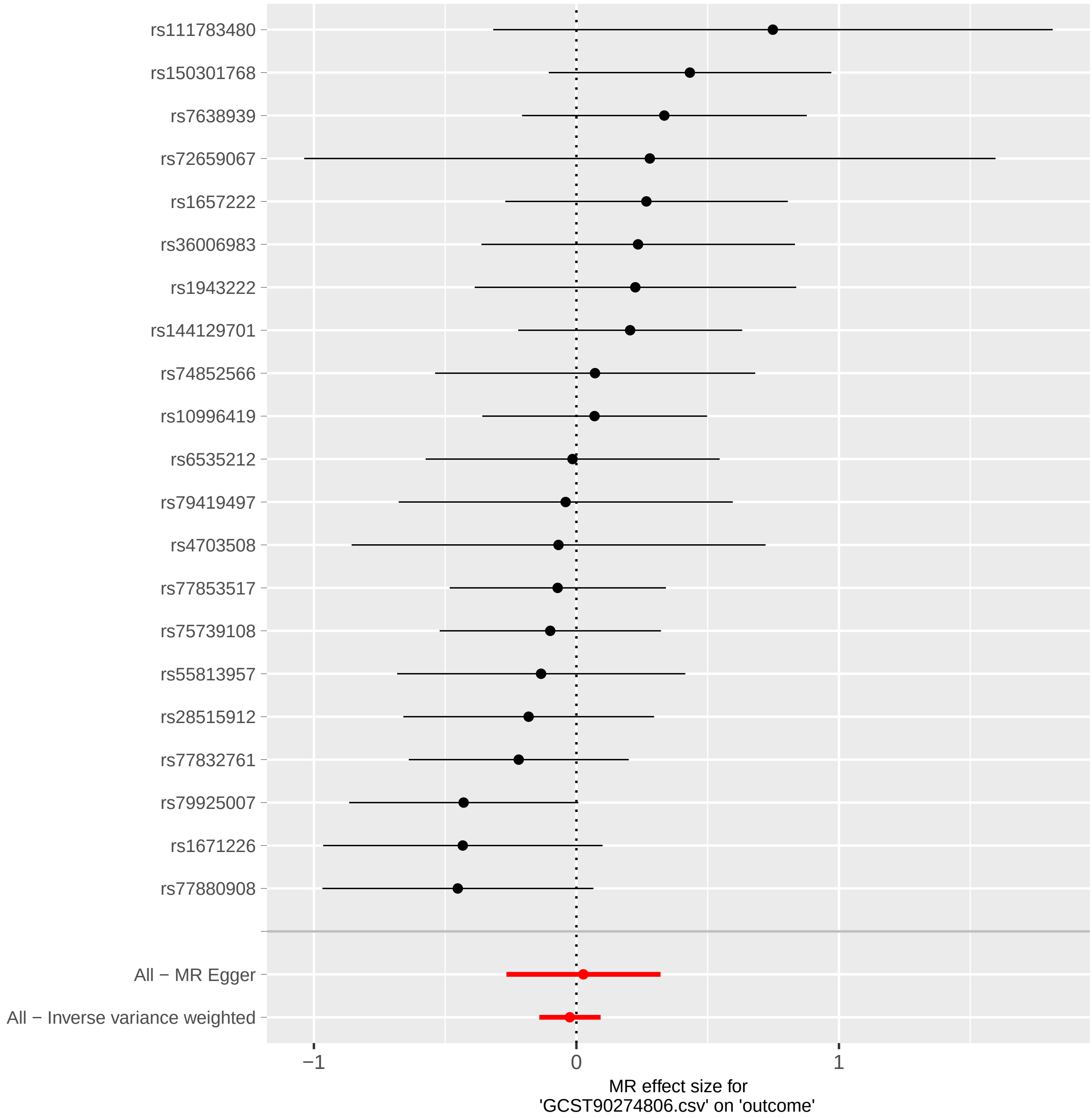
rs111783480
rs150301768
rs7638939
rs72659067
rs1657222
rs36006983
rs1943222
rs144129701
rs74852566
rs10996419
rs6535212
rs79419497
rs4703508
rs77853517
rs75739108
rs55813957
rs28515912
rs77832761
rs79925007
rs1671226
rs77880908
All − MR Egger
All − Inverse variance weighted
−1
0
1
MR effect size for
'GCST90274806.csv' on 'outcome'
